# Effect of Clove and Thyme Essential Oils on *Candida* Biofilm Formation and the Oil Distribution in Yeast Cells

**DOI:** 10.3390/molecules24101954

**Published:** 2019-05-21

**Authors:** Katarzyna Rajkowska, Paulina Nowicka-Krawczyk, Alina Kunicka-Styczyńska

**Affiliations:** 1 Institute of Fermentation Technology and Microbiology, Faculty of Biotechnology and Food Sciences, Lodz University of Technology, Wólczańska Str. 171/173, 90-924 Łódź, Poland; alina.kunicka@p.lodz.pl; 2 Department of Algology and Mycology, Faculty of Biology and Environmental Protection, University of Łódź, Banacha Str. 12/16, 90-237 Łódź, Poland; paulina.nowicka@biol.uni.lodz.pl

**Keywords:** food-borne *Candida* sp., biofilm, essential oils, anticandidal activity, confocal laser scanning microscopy

## Abstract

*Candida* biofilm structure is particularly difficult to eradicate, since biofilm is much more resistant to antifungal agents than planktonic cells. In this context, a more effective strategy seems to be the prevention of biofilm formation than its eradication. The aim of the study was to examine whether the process of initial colonization of materials (glass, polyethylene terephthalate, polypropylene) by food-borne *Candida* sp. can be impeded by clove and thyme essential oils, used at their minimal inhibitory concentrations. In the presence of clove oil, 68.4–84.2% of the yeast tested showed a statistically significant reduction in biofilm formation, depending on the material. After treatment with thyme oil, statistically significant decrease in biofilm cell numbers was observed for 63.2–73.7% of yeasts. Confocal laser scanning microscopy showed diverse compounds of clove and thyme oils that were disparately located in *C. albicans* cell, on a cell wall and a cell membrane, in cytoplasm, and in vacuoles, depicting the multidirectional action of essential oils. However, essential oils that were used in sub-inhibitory concentration were sequestrated in the yeast vacuoles, which indicate the activation of *Candida* defense mechanisms by cell detoxification. Clove and thyme essential oils due to their anti-biofilm activity can be efficiently used in the prevention of the tested abiotic surfaces colonization by *Candida* sp.

## 1. Introduction

Some species of the *Candida* genus are responsible for infections related to biofilm formation, which is considered to be an important virulence factor. Biofilm forming *Candida* strains are associated with higher patients mortality, probably correlated with the poor permeability of the matrix to the antifungal drugs [1]. Risk of *Candida* infections is associated not only with biofilm that is formed on medical devices, such as catheters, implants, endoprosthesis, fixation, and artificial valves, but also with the biofilm produced in food products and on food-packaging surfaces [2].

*Candida* biofilm is a heterogeneous, three-dimensional well-organized structure that consists of planktonic and mycelial yeast forms, surrounded by extracellular polymeric substances (EPS). The cells of the biofilm structure are interdependent in the quorum sensing system and they are characterized by different phenotypic properties from free-floating planktonic cells [3].

The structure of biofilm enhances the effectiveness of microbial protection against the adverse environmental factors, including antibiotics, reduces the effectiveness of host defense mechanisms and facilitates the acquisition of nutrients [4]. Biological mechanisms diminishing the sensitivity of the yeast biofilm to antifungal compounds are related to the active drug efflux, the limited drug penetration through the extracellular matrix, a low metabolic activity of cells in mature biofilm, and different expression of genes in biofilm than in planktonic forms [5,6]. The active drug efflux plays a principal role in the resistance in the early stage of biofilm development [6]. The role of the extracellular matrix in biofilm drug resistance seems to be less important, because the resistance occurs shortly after *C. albicans* adhesion to the surfaces, before EPS production [7]. The matrix function in the resistance is rather associated with the support of the biofilm integrity and the limitation of cells access to various compounds. Moreover, the EPS matrix does not affect the reduction of drug diffusion and the diffusion process is not dependent on the structure and maturity of the biofilm [8]. The drug resistance may also be a consequence of low metabolic activity and the slow growth of cells in a mature biofilm. However, the research on *C. albicans* biofilm with a controlled rate of growth show the relationship between drug resistance and the rate of growth only for the planktonic forms and not for biofilm phenotype [5]. An important attribute of biofilm is also the ability to produce a small population of persister cells that are extremely resistant to antifungal drugs [9]. Rather, the drug resistance of persister cells is the result of the metabolically dormant state of the cells and it is independent of cell membrane composition and efflux pump expression.

Due to *Candida* biofilm resistance to conventional antifungal therapy, efficient methods of treatment, as well as prophylaxis, need to be developed. In the light of findings regarding the high antifungal activity of essential oils [10], the question arises regarding their antibiofilm efficiency. Moreover, an important attribute of essential oils is their natural origin and the fact that, so far, the acquisition of resistance by microorganisms to essential oils was not yet observed.

The aim of the research was to examine whether the process of initial colonization of chosen abiotic surfaces (glass, polyethylene terephthalate, polypropylene) by food-borne *Candida* sp. and the formation of mature biofilm structure can be impeded by clove and thyme essential oils. In the study food-borne *Candida* strains of different origin were used, since, as we have shown previously, they exhibit some virulence factors and may be allocated in a group of risk of potential pathogens [11]. In addition, the similarity of food-borne and clinical strains implies the possibility of circulating of antibiotic-resistant strains outside the hospital environment and the possible yeast infection that is caused by yeasts entered via food [12]. Furthermore, in the study, observations with a confocal laser scanning microscope were performed to determine the distribution of essential oil in the yeast cell.

## 2. Results and Discussion

Candidiasis, which is most frequently caused by *C. albicans*, but also by *C. glabrata*, *C. tropicalis*, *C. krusei*, or *C. parapsilosis*, is often associated with the formation of biofilms on the surface of medical devices and tissues [13]. A wide range of biomaterials used in clinical practice has been shown to support colonization and biofilm formation by *Candida* spp. All of the materials tested in the study (glass, polypropylene, polyethylene terephthalate) are used in food packaging. Furthermore, polypropylene and polyethylene terephthalate are representing biomaterials that are used in implantable medical devices, i.e., heart valve structures, sutures, vascular grafts and prosthesis, shunt, and sutures [14].

**Figure 1:**
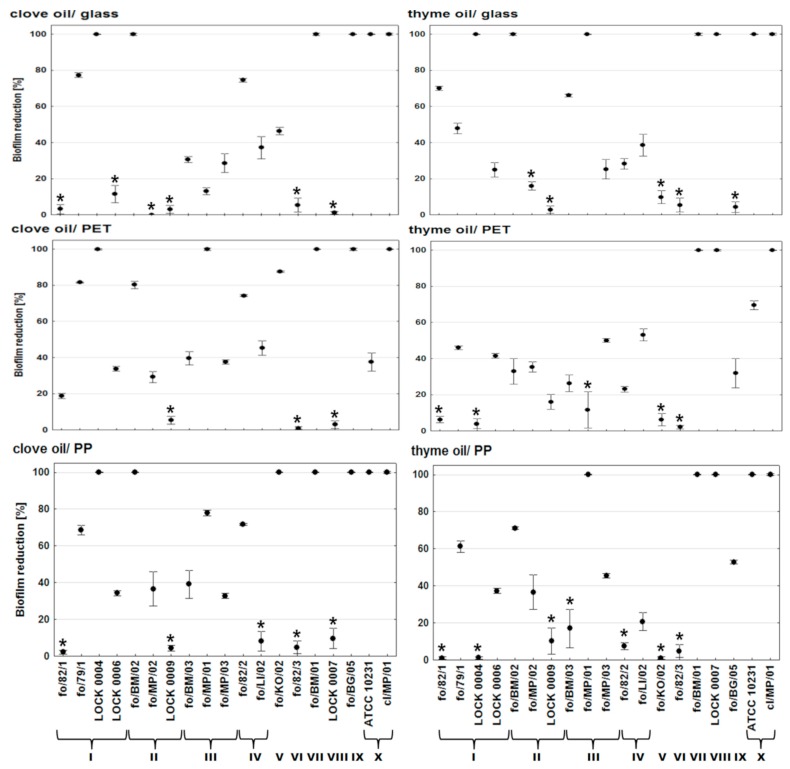
Percentage reduction in cell numbers of *Candida* biofilm on the surface of glass, polypropylene (PP) and polyethylene terephthalate (PET) in the presence of clove and thyme essential oils; *—no statistically significant reduction in comparison to control; I—*C. lusitaniae*, II—*C.* *krusei*, III—*C. boidinii*, IV—*C. famata*, V—*C. colliculosa*, VI—*C. parapsilosis*, VII—*C. tropicalis*, VIII—*C. pelliculosa*, IX—*C. rugosa*, X—*C. albicans*.

Essential oils were applicated at the early stage of biofilm formation, i.e., after adhesion phase, in order to determine whether thyme and clove oils can prevent the development of biofilm by *Candida* strains. The essential oils were used at MIC values that were determined for yeasts planktonic forms, appropriate for the oil and each strain. The treatment with clove oil resulted in the total inhibition of biofilm formation by *C. lusitaniae* LOCK 0004, *C. krusei* fo/BM/02, *C. tropicalis* fo/BM/01, *C. rugosa* fo/BG/05, and both *C. albicans* strains ATCC 10231 and cl/MP/01 on the surface of glass and polypropylene (PP). On PP, *C. colliculosa* isolate fo/KO/02 also did not develop biofilm (Figure 1). On polyethylene terephthalate (PET), clove oil completely inhibited biofilm formation by five strains *C. lusitaniae* LOCK 0004, *C. boidinii* fo/MP/01, *C. tropicalis* fo/BM/01, *C. rugosa* fo/BG/05, and *C. albicans* cl/MP/01. Only three isolates, *C. krusei* LOCK 0009, *C. parapsilosis* fo/82/3, and *C. pelliculosa* LOCK 0007, showed resistance to clove oil in terms of biofilm formation on all of the materials tested. Except for complete inhibition of biofilm development in the presence of clove oil, the number of cells in the biofilm structure was reduced on a statistically significant level in 53.8% yeasts on the surface of glass (by 13.1–77.3%), 78.6% on PET (18.8–87.5%), and 58.3% on PP (34.3–77.9%).

After treatment with thyme oil, the total inhibition of biofilm development was observed for three strains (*C. tropicalis* fo/BM/01, *C. pelliculosa* LOCK 0007, and *C. albicans* cl/MP/01) on all of the materials tested (Figure 1). Additionally, *C. albicans* ATCC 10231 and *C. boidinii* fo/MP/01 on the surface of glass and PP, and also *C. lusitaniae* LOCK 0004 and *C. krusei* fo/BM/02 on glass, did not grow in a biofilm structure. Furthermore, in the presence of thyme oil 58.3% isolates showed a statistically significant decrease in cells number in biofilm on the surface of glass (by 24.9–66.1%), 68.8% on PET (by 16.0–69.5%), and 50% on PP (by 20.7–71.0%). Only two yeast *C. colliculosa* fo/KO/02 and *C. parapsilosis* fo/82/3 were insensitive to thyme oil in the concentrations used, which resulted in biofilm formation on the materials under study (Figure 1).

**Figure 2:**
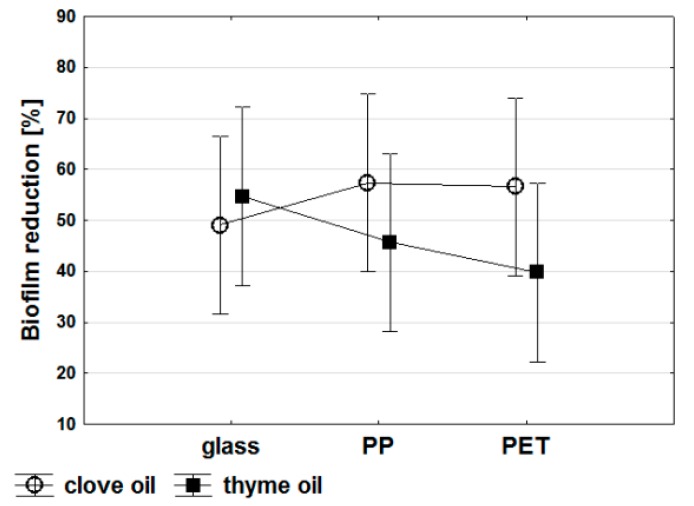
Multivariate analysis of variance (MANOVA) for activity of clove and thyme oils against *Candida* biofilm development on abiotic surfaces (glass, polypropylene PP, polyethylene terephthalate PET).

Multivariate analysis of variance (MANOVA) was used to find the manner in which the independent variables (the type of abiotic surface and the type of essential oil) affected the collectively treated set of dependent variables (reduction in biofilm formation by 19 *Candida* strains tested). On the basis of MANOVA analysis, no statistically significant direct relationship was found between the type of oil, as well as the material and their effect on biofilm formation. However, some dependencies can be determined. Generally, biofilm development was limited more by clove than thyme oil on the surface of PP and PET (Figure 2). Clove oil exhibited the highest antibiofilm efficiency on PP, whereas the lowest on glass. Thyme oil was the most effective in protecting the surface of glass from biofilm formation by *Candida* sp. (Figure 2). Furthermore, on the surface of glass, both of the oils limited the formation of biofilm in a comparable range. More significant differences (about 10%) concerned the antibiofilm activity of clove and thyme oils on the surface of PP. Whereas, the impact of both oils on biofilm reduction on the surface of PET varied by 15 to 20%. The antibiofilm effectiveness of the essential oils tested can be related to the physicochemical properties of abiotic materials, since microbial adhesion preferably occurs on hydrophobic surfaces than on hydrophilic ones with lower contact angles. Polymers, such as PP or PET with a higher contact angle (108° and 103°, respectively), are more hydrophobic than glass (38.5°) [15,16]. Another important factor can be substratum surface roughness, which enhances the rate of biofilm adhesion when increased. Glass is characterized by very smooth surface and higher ease of the relative biofilm removal when compared to polymers [16].

To date, the methods that were used or developed to eradicate biofilms in industrial conditions can be classified into three groups: physical (mechanical surface cleaning, exposure to high or low temperature, use of ultrasonic waves, electric field or cold plasma, application of photodynamic therapy), biological (matrix-targeting enzymes, phagotherapy), and chemical (drugs, biocides, surfactants) [17]. Due to the fact that these methods of biofilm eradications are time-consuming and often ineffective—a better strategy seems to be prevention of biofilm formation.

In this context, the application of essential oils could be promising in reducing the risk of biofilm development. Especially that, according to presented results, at the early stage of biofilm formation, essential oils can be used, even at MIC concentration, usually much lower than the minimum biofilm inhibitory concentration. Khan and Ahmad [18] reported the high inhibitory effect of *Cymbopogon citratus* and *Syzygium aromaticum* oils on biofilm formation by drug-resistant *Candida* spp., as related to the inhibition of filamentation, matrix disappearance, destruction of three-dimensional structure of biofilm, and shrinkage in the cell membranes of sessile cells. Likewise, eucalyptus, peppermint, ginger grass, and clove oils have been shown to reduce *C. albicans* biofilm formation by 28.6–80.9%, probably by exerting a metabolic interference in *Candida* biofilm [19]. *Salvia officinalis* essential oil at MIC and sub-MIC concentrations can significantly reduce *C. albicans* adhesion to polymethyl methacrylate resin surface, and thus can be used as an antifungal denture cleanser [20]. Strong antibiofilm activity have been also reported for *Thymus vulgaris* and *Carum copticum* oils against *Candida* spp. of various clinical origin, and their effectiveness, even at sub-MICs, was much higher than fluconazole [21].

The action of essential oils, due to their complexity, is usually considered as a synergistic effect of a few main compounds. Although the possible mode of action of the particular essential oils main components against *C. albicans* is still under research, there are some works that underlie the role of eugenol, thymol, and carvacrol in the inhibition of H^+^-ATPase and efflux pumps activity [22] or *Mentha suaveolens* essential oil—composed of linalool and borneol among others—in biofilm restriction [23]. A substantial decrease in *C. albicans* cell adherence was observed after thymol and eugenol treatment [24]. Moreover, linalool and α-pinene, which are the components of thyme oil, are proved to have an antifungal effect against *Pichia anomala* yeast and anti-quorum sensing effect by violating *N*-acylhomoserine lactone (AHL) expression in *Chromobacterium violaceum* [25].

The preservation of *Candida* biofilm development is of paramount importance in nosocomial environments, since biofilm formation was reported as an independent predictor of increased mortality [26]. *Candida* species in the form of biofilm associated with food may pose a threat to a massive influx into the human intestinal tract. The risk of candidiasis interlinked with food-borne strains is related to the possibility of yeasts penetration into human oral epithelial cells [27] and persorption, even through undamaged mucosa [28]. Biofilm formation in the food industry may also cause high economic losses. The trends in antibiofilm strategies involve the modification of material surface to reduce the adhesion of microorganisms and interference in the initial phase of biofilm development. To date, some antimicrobial or anti-adhesion coatings have been successfully implemented on materials to avoid microbial colonization, including furanones coated silicone, polypropylene, and polytetrafluorethylene, oregano essential oil introduced into polylactic acid films, or poly(ethylene oxide) with encapsulated tea tree oil and beta-cyclodextrin [29,30,31]. The application of essential oils in materials coatings can provide both anti-adhesive and anticandidal effects. However, further research should be carried out in this area.

**Figure 3:**
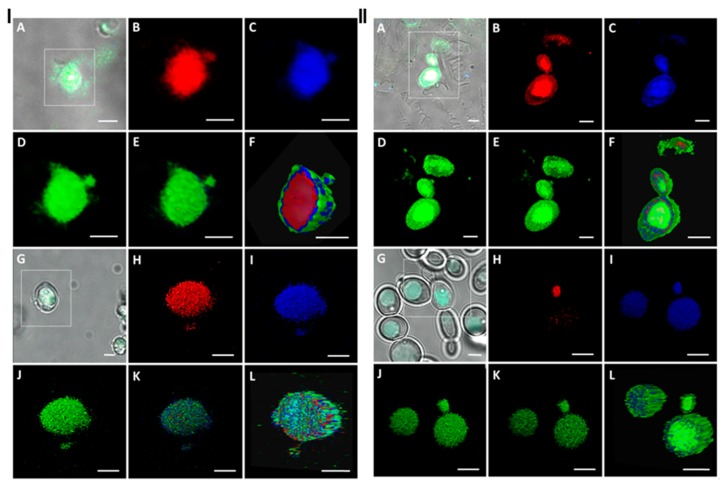
The three-dimensional Confocal Laser Scanning Microscope (3D CLSM) images of *C. albicans* ATCC 10321 under the clove oil (I) and thyme oil (II) treatment, scale bar 2 µm. Four-channel fluorescence of thyme/clove oil at MIC: (**A**) Transmission Channel, (**B**) Red Channel, (**C**) Blue Channel, (**D**) Green Channel, (**E**) stack of Red-Blue-Green Channels, (**F**) cross-section of Red-Blue-Green Channels stack (green fluorescence on the cell surface, blue and red fluorescence in the inner part of the cell in cytoplasm, all fluorescence signals in vacuoles). Four-channel fluorescence of thyme/clove oil at ½ MIC: (**G**) Transmission Channel, (**H**) Red Channel, (**I**) Blue Channel, (**J**) Green Channel, (**K**) stack of Red-Blue-Green Channels, (**L**) cross-section of Red-Blue-Green Channels stack (green, blue and red fluorescence signals in vacuoles).

Apart from the inhibitory activity of the essential oils, their antibiofilm action is also presumably linked with the oil location within the yeast cells and it may have an effect on the interactions between the cell and the abiotic surface. We used a confocal laser scanning microscopy technique to visualize the clove and thyme oils placement in the *Candida* yeast cell. This technique allows for tracing plant secondary metabolites distribution with both the plant and microorganism cells due to the autofluoresescence phenomenon [32,33]. This model study was conducted with the use of the reference *C. albicans* ATCC 10231 strain, and essential oils in two concentrations, i.e., MIC, as in evaluation of biofilm development, and ½ MIC to assess the effect of essential oils in the sub-inhibitory concentration. Excitation by 405 nm resulted in an emission of fluorescence being detected in three channels: at 430–480 nm in a Blue Channel, at 500–550 nm in a Green Channel, and at 600–650 nm in a Red Channel, responding to the respective colour images (Figure 3).

The clove and thyme oils differed in chemical compositions, although both of the essential oils are characterized by a high contents of phenolic compounds (Table 1). Regardless of the type and chemical nature of the essential oil, the localization of both oils in yeast cell was similar. At MIC concentrations of the oils, their compounds disparately diffused into the cell (Figure 3). A green fluorescence was visible on the cell wall surface, indicating that the oil compounds covered the surface of the cell or adhered to the external cell structures, i.e., cell wall or cellular membrane. A blue fluorescence signal has shown the oil compounds penetrating into the cell and probably localized into the cytoplasm (Figure 3, I F and II F). Similarly, the red fluorescence signal was noticeable in the cytoplasm. Moreover, three fluorescence signals were detected in the inner part of the cell in vacuoles (Red, Blue, and Green Channels superimposed) (Video S1 and S3), indicating the presence of the oils compounds. Interestingly, after the yeast treatment with clove and thyme oils at ½ MIC concentrations, all of the detected signals were localized in vacuoles, and the weakest one was registered in the Red Channel (Figure 3, Video S2 and S4). Besides vacuoles, no fluorescence inside the *C. albicans* cell or on the surface of the cell was detected. The vacuolar localization of the essential oils indicates the action of cell defense mechanisms against essential oils by isolating them within the cell and limiting contact with cell organelles.

For essential oils, the spectral interference was noted due to their complex multicomponent nature [32,34]. The bright fluorescence of both terpenoids and phenols in blue or blue-green was recorded in a variety plant cells [32]. The blue and green fluorescence signals that were detected in the *C. albicans* cells may be mainly attributed to the terpene eugenol and the terpenoid phenol thymol, prevailing compounds of the clove and thyme essential oils, respectively.

Previously we have reported that various essential oils of different chemical composition affected the same cellular targets, and the cytotoxic and genotoxic effects occur by the same universal mechanism [35]. This assumption was confirmed in this study, and probably the activity of the various essential oils resulted from their complex nature rather than the presence of a particular compound. As we have shown earlier, the anticandidal activity of thyme and clove oils is related to the disruption of the permeability barrier of *C. albicans* cell membrane structures [35]. This finding, together with CLSM observations in the present study, suggests two explanations for the oils different distribution in the cell when used at sub-MIC and MIC concentrations. Probably, at the lower oil concentration, all of its compounds are separated in the yeast cell vacuole, whereas at the higher concentration of the oil this mechanism of a cell defense is not efficient enough. However, the possibility that the oil treatment at the MIC concentration leads to the loss of membranes integrity cannot also be excluded, resulting in the release of essential oils compounds, which were initially located in vacuoles, into other cell compartments.

Ramsay and Gadd [36] and Cornelius and Nakashima [37] demonstrated the role of vacuoles in detoxifying the cytoplasm by sequestering e.g., heavy metal ions or calcium ions at toxic concentration. Yeast vacuoles are the largest lysosomes-like organelle in fungi, participating in the degradation of cell components, storage of ions, metabolites, and organic and inorganic nutrients. Vacuoles play a key role in homeostasis, which is important for adaptation to new environments and survival under stressful conditions [38]. Moreover, in *C. albicans,* the vacuole is crucial for the formation of the tissue invasive hyphal form, and it is required for virulence [39].

In conclusion, clove and thyme oils can be used as effective anti-biofilm agents in the prevention of materials colonization by *Candida* sp. Thus, a valuable direction for further research could be the application of these oils as materials coatings to prevent *Candida* biofilm formation. In addition, yeast seems to be able to actively carry out the detoxification process involving vacuoles, when treated with essential oils in sub-lethal concentrations. To our best knowledge, this is the first report regarding defence mechanisms of yeast cell against the toxic effects of essential oils.

## 3. Materials and Methods

### 3.1. Essential Oils

The antibiofilm activity against food-borne *Candida* strains was estimated for clove (*Syzygium aromaticum* (L.) Merr. & L.M. Perry) and thyme (*Thymus vulgaris* L.) essential oils. The commercial oils that were purchased from Pollena Aroma S.A. (Warszawa, Poland) were used. Chemical composition of EOs was analyzed by GC-MSFID using Trace GC Ultra (Thermo Scientific, Waltham, MA, USA) combined with DSQ II mass spectrometer and with flame ionization detector (FID) throughout the MS-FID splitter (SGE; Analytical Science, Trajan, Australia). Analysis was provided using a nonpolar capillary column Rtx-1 ms (60 m × 0.25 mm, with a film thickness 0.25 µm; Restek, Bellefonte, PA, USA). The oven temperature was programmed, as follows: 50–300 °C at 4 °C/min; injector temperature 280 °C; detector temperature 310 °C; carrier gas helium with regular pressure 200 kPa; and, ionization energy 70 eV, ion source temperature 200 °C. The identification of components was based on the comparison of their mass spectra with those in a laboratory-made MS library, commercial libraries (NIST 09, Wiley 275.1, Mass Finder 4), along with the retention indices that were associated with a series of alkanes with linear interpolation (C8–C26). A quantitative analysis (expressed as percentages of each component) was carried out by peak area normalization measurements without correction factors. Table 1 presents the chemical composition of EOs.

### 3.2. Yeasts

In the study, 17 food-borne *Candida* isolates (Table 2) and one clinical strain *C. albicans* cl/MP/01 (obtained from Department of Laboratory Diagnostics of Polish Mother’s Memorial Hospital—Research Institute in Lodz, Poland) were examined. As a reference *C. albicans* ATCC 10231 isolated from a man with bronchomycosis was used.

### 3.3. Biofilm Formation

The influence of clove and thyme oils on biofilm formation was tested on different surfaces (glass, polypropylene PP, polyethylene terephthalate PET), as described previously [11]. Briefly, the *Candida* strains were grown in liquid Sabouraud medium (Merck KGaA, Darmstadt, Germany) and incubated at 30 °C for 24 h. 2 mL of *Candida* cell suspensions (10^6^ cells/mL) were inoculated onto the material slices (20 mm × 20 mm) that were contained in six-well tissue plates (Greiner Bio-One GmbH, Frickenhausen, Germany) and allowed to adhere for 90 min. at 30 °C in an orbital shaker (75 rpm) (adhesion phase). The non-adherent cells were then removed by gently washing twice with 1 mL PBS and essential oil at MIC concentration (Table 3) in liquid Sabouraud medium was added. The minimal inhibitory concentrations of clove and thyme oils were estimated previously [10] by the means of the broth macrodilution method. Afterwards, the plates were incubated at 30 °C for 48 h (biofilm formation phase). Material slices with adhered *Candida* cells were incubated in Sabouraud broth without addition of essential oils in the controls. Quantification of biofilm was performed by the standard count plate method and the number of yeast was determined on Sabouraud dextrose agar (Merck KGaA, Darmstadt, Germany). The plates were incubated for 48 h at 30 °C.

The results of the analysis are presented as a reduction in the number of yeast in biofilm structure calculated from the formula:

Reduction [% (CFU/cm^2^)] = (log B − log A)/log B × 100

where: A—number of cells in biofilm in the presence of essential oil [CFU/cm^2^], B—number of cells in biofilm structure without the addition of essential oil [CFU/cm^2^].

### 3.4. Observations using Confocal Laser Scanning Microscope (CLSM)

Cells of *C. albicans* ATCC 10321 that were treated with essential oils were examined using the Leica TCS SP8 Confocal Laser Scanning Microscope (Leica Microsystems, Wetzlar, Germany) and the LAS X 2.0.2.15022 software (Leica Microsystems, Wetzlar, Germany) in the Laboratory of Microscopic Imaging and Specialized Biological Techniques, University of Łódź. The fluorescence excitation of oils was induced using UV (Leica Microsystems, Wetzlar, Germany) diode of 405 nm., while the detection was recorded by three separate detectors with 800 ± 5 gain and -0.5 offset: PMT 1 at a 430–480 nm (Blue Channel), PMT 3 at a 500–550 nm (Green Channel), and PMT 5 at a 600–650 nm (Red Channel). Moreover, a PMT Trans Channel was used to visualize the cells in transmitted light. Each sample was scanned in the ‘xyz’ axes to a depth of 30 µm (three-dimensional (3D) scan) while using HC PL APO CS2 63×/1.40 (Leica Microsystems, Wetzlar, Germany) Oil objective. Line Average of 4× was used to improve the quality of images. The images were visualised in 3D view using a surface and blend mode with an adjusted threshold for a cross-section of cells.

### 3.5. Statistical Analysis

Statistical calculations were carried out with the use of Statistica v.10.0 software package (StatSoft. Inc., Tulsa, OK, USA). All of the results were expressed as the mean ± SD of three independent experiments. One-way ANOVA test was performed in order to compare the statistical differences (*p* < 0.05) in biofilm formation and multivariate analysis of variance MANOVA was used for the simultaneous examination of relationship between independent variables of the qualitative type (type of essential oil and type of material) and the dependent variables of the quantitative type (biofilm reduction).

## Figures and Tables

**Table 1:** The main components (≥0.4%) of examined essential oils (GC-MS analysis).

Compound	RI ^1^	Clove Oil	Thyme Oil
		Content [%]	
α-Thujene	926	−	0.9
α-Pinene	934	−	0.9
Campehen	940	−	0.4
β-Myrcene	983	−	1.8
Car-3-ene	1008	−	2
*p*-Cymene	1016	−	18.4
β-Phellandrene	1019	−	0.4
Limonene	1025	−	0.9
γ-Terpinene	1055	−	8.8
Linalool	1086	−	3.2
Borneol	1155	−	0.7
Thymol	1281	−	48.6
Carvacrol	1285	−	5.5
Eugenol	1342	85.2	−
(*E*)-β-Caryophyllene	1421	9.9	2.3
α-Humulene	1453	1.9	0.1
δ-Cadinene	1513	0.4	0.2
(*E*)-β-Caryophyllene oxide	1568	0.4	0.4

^1^ RI, retention index.

**Table 2:** Origin of food-borne isolates tested.

Species	Isolate	Origin
*C. lusitaniae*	fo/82/1, fo/79/1	fruit yoghurt
	LOCK 0004, LOCK 0006	feed
*C. krusei*	fo/BM/02, fo/MP/02	pickled cucumber
	LOCK 0009	bakery
*C. boidinii*	fo/BM/03, fo/MP/01, fo/MP/03	pickled cucumber
*C. famata*	fo/82/2	fruit yoghurt
	fo/LI/02	herring salad
*C. colliculosa*	fo/KO/02	fruit yoghurt
*C. parapsilosis*	fo/82/3	fruit yoghurt
*C. tropicalis*	fo/BM/01	pickled cucumber
*C. pelliculosa*	LOCK 0007	feed
*C. rugosa*	fo/BG/05	sauerkraut

**Table 3:** Anti-*Candida* activity of clove and thyme essential oils at MIC values.

Strain	Clove Oil	Thyme Oil
	Concentration [% *v*/*v*]	
*C. lusitaniae* fo/82/1	0.12	0.06
*C. lusitaniae* fo/79/1	0.25	0.12
*C. lusitaniae* LOCK 0004	0.12	0.12
*C. lusitaniae* LOCK 0006	0.06	0.12
*C. krusei* fo/BM/02	0.12	0.12
*C. krusei* fo/MP/02	0.06	0.03
*C. krusei* LOCK 0009	0.12	0.12
*C. boidinii* fo/BM/03	0.12	0.25
*C. boidinii* fo/MP/01	0.12	0.12
*C. boidinii* fo/MP/03	0.06	0.03
*C. famata* fo/82/2	0.12	0.03
*C. famata* fo/LI/02	0.03	0.03
*C. colliculosa* fo/KO/02	0.12	0.12
*C. parapsilosis* fo/82/3	0.06	0.06
*C. tropicalis* fo/BM/01	8	8
*C. pelliculosa* LOCK 0007	0.06	0.06
*C. rugosa* fo/BG/05	0.12	0.12
*C. albicans* ATCC 10231	0.25	0.25
*C. albicans* cl/MP/01	0.25	0.5

## Supplementary Materials

The following are available online. Video S1: A confocal microscopy 3D movie showing *C. albicans* ATCC 10231 cell treated with clove oil at MIC concentration, as shown in Figure 3I-F; Video S2: A confocal microscopy 3D movie showing *C. albicans* ATCC 10231 cell treated with clove oil at ½ MIC concentration, as shown in Figure 3I-L; Video S3: A confocal microscopy 3D movie showing *C. albicans* ATCC 10231 cell treated with thyme oil at MIC concentration, as shown in Figure 3II-F; Video S4: A confocal microscopy 3D movie showing *C. albicans* ATCC 10231 cell treated with thyme oil at ½ MIC concentration, as shown in Figure 3II-L.
